# Supplementary material for: Exploring Heterogeneity of Fecal Microbiome in Long COVID Patients at 3 to 6 Months After Infection
Source: Int J Mol Sci. 2025 Feb 19;26(4):1781. doi: 10.3390/ijms26041781 (PMC11855614; doi:10.3390/ijms26041781)
Supplement: Supplementary file 1 [file ijms-26-01781-s001.zip › On behalf of P4O2 v6.pdf]

On behalf of P4O2

M.I. Abdel-Aziz, Amsterdam UMC, Amsterdam, The Netherlands, and Assiut University, Assiut, Egypt; N. Baalbaki, Amsterdam UMC, Amsterdam, The Netherlands; S. Bazdar, Amsterdam UMC, Amsterdam, The Netherlands; I. Beekers, Clinical Care & Research department, ORTEC, Zoetermeer, The Netherlands; R.J.H.C.G. Beijers, Maastricht University Medical Centre+, Maastricht, The Netherlands; J.M. Blankestijn, Amsterdam UMC, Amsterdam, The Netherlands; M. van de Berge, GRIAC Research Institute, Groningen, The Netherlands; J.P. van den Bergh, VieCuri Medical Center, Venlo, The Netherlands; L.D. Bloemsma, Amsterdam UMC, Amsterdam, The Netherlands; H.J. Bogaard, Amsterdam UMC, Amsterdam, The Netherlands; C.D.C. Born, Maastricht University Medical Centre+, Maastricht, The Netherlands; M.S.G.M. Bracke, NetherlandsClear.bio, Clear. BV, Amsterdam, The Netherlands; M.E.B. Cornelissen, Amsterdam UMC, Amsterdam, The Netherlands; A.M. van der Does, Leiden University Medical Center, Leiden, the Netherlands; G.S. Downward, Institute for Risk Assessment Sciences (IRAS), Utrecht University, and Julius Center for Health Sciences and Primary Care, University Medical Center Utrecht, Utrecht, C.M. van Drunen, Amsterdam UMC, Amsterdam, The Netherlands; The Netherlands; J.W. Duitman, Amsterdam UMC, Amsterdam, The Netherlands; D. Gach, Maastricht University Medical Centre+, Maastricht, The Netherlands; J. Garssen, Utrecht Insitute for pharmaceutical sciences, Utrecht, the Netherlands; J.J.M. Geelhoed, Leiden University Medical Center, Leiden, the Netherlands; J. Glastra, Quantib, Rotterdam, The Netherlands; K. Golebski, Amsterdam UMC, Amsterdam, The Netherlands; I.H. Heijink, GRIAC Research Institute, Groningen, The Netherlands; P.S. Hiemstra, Leiden University Medical Center, Leiden, the Netherlands; J.C.S. Holtjer, Institute for Risk Assessment Sciences, Utrecht University, Utrecht, The Netherlands; S. Holverda, Lung Foundation Netherlands, Amersfoort, The Netherlands; L. Houweling, Institute for Risk Assessment Sciences, Utrecht, and Amsterdam UMC, Amsterdam, The Netherlands; J.J.L. Jacobs, Clinical Care & Research department, ORTEC, Zoetermeer, The Netherlands; P.A. de Jong, UMC Utrecht, Utrecht; R. Jonker, Amsterdam UMC, Amsterdam, The Netherlands; P.J.M. Kuks, GRIAC Research Institute, Groningen, The Netherlands; A.K.A.L. Kwee, University Medical Center Utrecht, Utrecht, The Netherlands; R.C.J. Langen, Maastricht University Medical Centre+, Maastricht, The Netherlands; P.M.A. Linders, Amsterdam UMC, Amsterdam, The Netherlands; A.H. Maitland-van der Zee, Amsterdam UMC, Amsterdam, The Netherlands; P. Moeskops, Quantib, Rotterdam, The Netherlands; F.A.A. Mohamed Hoesein, University Medical Center Utrecht, Utrecht, The Netherlands; J. Mosayebi Amroabadi, Maastricht University Medical Centre+, Maastricht, The Netherlands; L. Noij, Amsterdam UMC, Amsterdam, The Netherlands; E.J. Nossent, Amsterdam UMC, Amsterdam, The Netherlands. J. Olsson, Smartfish AS, Oslo, Norway; J. Otker, Patient representative Lung Foundation Netherlands, Amersfoort, The Netherlands; S.D. Pouwels, GRIAC Research Institute and UMCG, Groningen, The Netherlands; B.R. Rae, GRIAC Research Institute, Groningen, The Netherlands; L.B. Richards, Clinical Care & Research department, ORTEC, Zoetermeer, The Netherlands; A. Rodriguez Ruiz, Leiden University Medical Center, Leiden, the Netherlands; D.W. Schaminee, Amsterdam UMC, Amsterdam, The Netherlands; A.M.W.J. Schols, Maastricht University Medical Centre+, Maastricht, The Netherlands; L.T. Schuurman, Maastricht University Medical Centre+, Maastricht, The Netherlands; B.M. Sondermeijer, Spaarne Gasthuis, Haarlem, The Netherlands; I. Verkouter, Clinical Care & Research department, ORTEC, Zoetermeer, The Netherlands R. de Vries, Breathomix B.V., Leiden, The Netherlands; Y. de Wit-van Wijck, Amsterdam UMC, Amsterdam, The Netherlands.

Conflict of interest:

M.I. Abdel-Aziz has nothing to declare. N. Baalbaki has nothing to declare. S. Bazdar has nothing to declare. I. Beekers has nothing to declare. R.J.H.C.G. Beijers has nothing to declare. J.M. Blankestijn has nothing to declare. M. van de Berge has nothing to declare. J.P. van den Bergh has a research funding from UCB and Amgen (outside P4O2). L.D. Bloemsma has nothing to declare. H.J. Bogaard received a Grant support Ferrer (Janssen, MSD). C.D.C. Born has nothing to declare. M.S.G.M. Bracke is the Co-founder of Clear. M.E.B. Cornelissen has nothing to declare. A.M. van der Does has nothing to declare. G.S. Downward has nothing to declare. C.M. van Drunen has nothing to declare. J.W. Duitman received financial support from Boehringer and Abbvie for the P4O2 ILD cohort. D. Gach has nothing to declare. J. Garssen has nothing to declare. J.J.M. Geelhoed has nothing to declare. J. Glastra is employee at Quantib. K. Golebski has nothing to declare. I.H. Heijink has a Research grant from Boeheringer Ingelheim outside the scope of the submitted work. P.S. Hiemstra has nothing to declare. J.C.S. Holtjer has nothing to declare. S. Holverda has nothing to declare. L. Houweling has nothing to declare. J.J.L. Jacobs has nothing to declare. R. Jonker has nothing to declare. P.J.M. Kuks has nothing to declare. A.K.A.L. Kwee has nothing to declare. R.C.J. Langen has nothing to declare. P.M.A. Linders has nothing to declare. A.H. Maitland-van der Zee has received research grants outside the submitted work from GSK, Boehringer Ingelheim, AbbVie and Vertex, she is the PI of P4O2 (Precision Medicine for more Oxygen), a public private partnership co-funded by Health~Holland involving many private partners that contribute in-cash and/or in-kind (Abbvie, Aparito, Boehringer Ingelheim, Breathomix, Clear, Danone Nutricia Research, Fluidda, Ncardia, Olive, Ortec Logiqcare, Philips, Quantib, RespiQ, Roche, Smartfish, SODAQ, Thirona, TopMD and Novartis), and she has served in advisory boards for AstraZeneca, GSK, and Boehringer Ingelheim with money paid to her institution. P. Moeskops is employee at Quantib. F.A.A. Mohamed Hoesein has nothing to declare. J. Mosayebi Amroabadi has nothing to declare. L. Noij has nothing to declare. E.J. Nossent has speaker fees for lectures and educational events (Janssen, Bayer/MSD, Astra Zeneca) United Therapeutics/Ferrer, Boehringer Ingelheim B.V., Chiesi. J. Olsson is employed at Smartfish which produce Remune (which is used in P4O2 as oral nutrition product). J. Otker has nothing to declare. S.D. Pouwels has nothing to declare. B.R. Rae has nothing to declare. L.B. Richards has nothing to declare. A. Rodriguez Ruiz has nothing to declare. D.W. Schaminee has nothing to declare. A.M.W.J. Schols has nothing to declare. L.T. Schuurman has nothing to declare. S. Shahbazi Khamas has nothing to declare. B.M. Sondermeijer has nothing to declare I. Verkouter has nothing to declare. R. de Vries receives personal fees and has a substantial interest in the company Breathomix BV. Y. de Wit-van Wijck has nothing to declare.
