# Supplementary material for: Exploring Heterogeneity of Fecal Microbiome in Long COVID Patients at 3 to 6 Months After Infection
Source: Int J Mol Sci. 2025 Feb 19;26(4):1781. doi: 10.3390/ijms26041781 (PMC11855614; doi:10.3390/ijms26041781)
Supplement: Supplementary file 1 [file ijms-26-01781-s001.zip › P4O2_fecal_microbiome_supplements.pdf]

Supplementary information

Table S1: 15 butyrate producing genera that were used to determine the percentage of butyrate producing bacteria in our patient population.

**Genus**
Butyricimonas
Odoribacter
Anaerostipes
Anaerobutyricum
Agathobacter
Butyrivibrio
Coprococcus
Roseburia
Shuttleworthia
Butyricicoccus
Faecalibacterium
Flavonifractor
Pseudoflavonifractor
Oscillibacter
Subdoligranulum

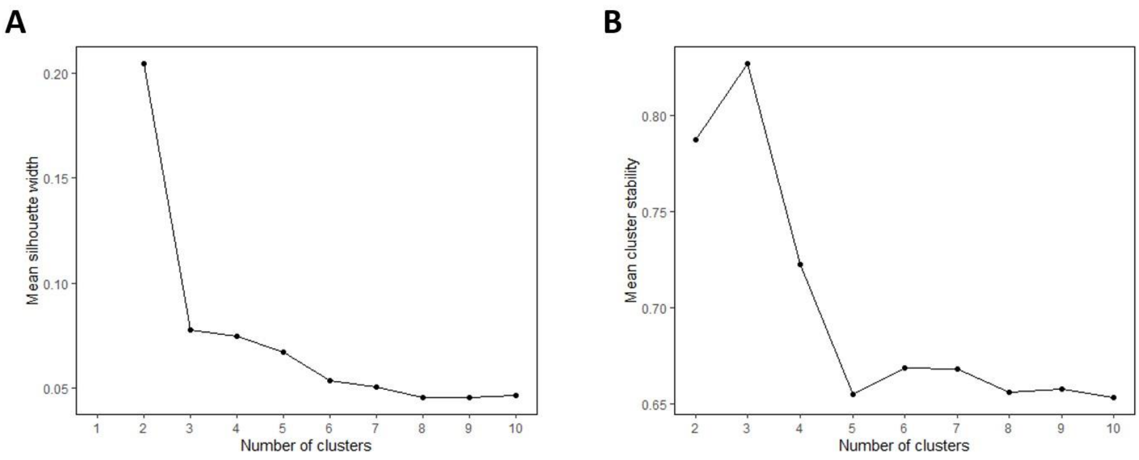

Figure S1 cluster validity indices*: plots showing the cluster validity measures for the unsupervised clustering. Part A shows the mean silhouette index, while part B shows the mean cluster stability based on bootstrapping.*

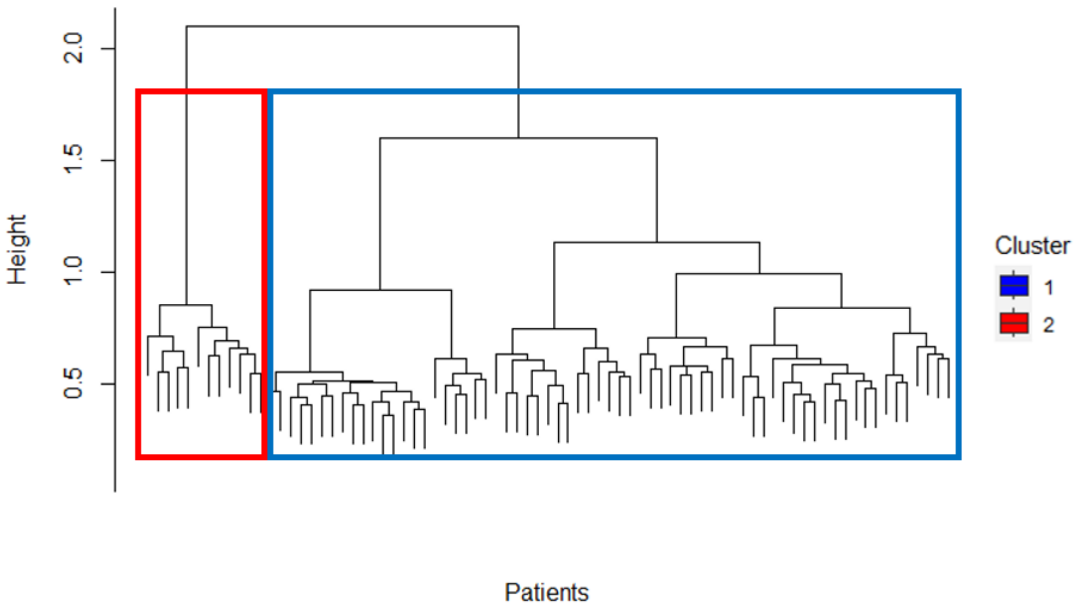

***Figure S2 cluster dendrogram**: Dendrogram from the unsupervised hierarchical clustering.*

Table S1: Patient characteristics of only the patients admitted to the ICU, according to cluster. Numerical data is shown as mean ± sd or median (IQR) while categorical data is shown as n/N (%). *P-values are calculated using either a Wilcoxon signed-rank test or Student's t-test for numerical data, or a Fisher's exact test for categorical data.*

	**1 (N = 15)**	**2 (N = 11)**	**p-value**
**General Characteristics**			
Sex (Female)	6/15 (40.0%)	3/11 (27.3%)	0.683
Age (Years)	55.6 ± 5.4	55.2 ± 6.3	0.857
BMI (kg/m^2)	29.3 ± 2.6	30.6 ± 6.6	0.511
Time since SARS-CoV-2 infection (days)	173.0 (125.5, 193.0)	167.0 (147.0, 215.0)	0.622
**Co-morbidities**			
Asthma	3/15 (20.0%)	3/11 (27.3%)	1.000
COPD	1/15 (6.7%)	0/11 (0.0%)	1.000
CVD	3/14 (21.4%)	3/11 (27.3%)	1.000
Diabetes	3/15 (20.0%)	2/11 (18.2%)	1.000
**Symptom Categories**			
Fatigue	9/15 (60.0%)	7/11 (63.6%)	1.000
Respiratory	10/15 (66.7%)	11/11 (100.0%)	0.053
Neurological	10/15 (66.7%)	8/11 (72.7%)	1.000
Cardiovascular	5/15 (33.3%)	1/11 (9.1%)	0.197
Gastrointestinal	3/15 (20.0%)	2/11 (18.2%)	1.000
Other	1/15 (6.7%)	4/11 (36.4%)	0.128
**Lung Function**			

	1 (N = 15)	2 (N = 11)	p-value
FEV1 (% predicted)	93.2 ± 11.2	71.7 ± 15.3	**< 0.001**
FVC (% predicted)	87.7 ± 12.0	68.1 ± 18.8	**0.003**
FEV1/FVC (%)	82.9 ± 4.7	83.3 ± 8.5	0.897
DLCO (% predicted)	77.6 ± 13.1	56.7 ± 20.7	**0.004**
**Questionnaires***			
FSS (↓)	3.9 (2.4, 5.9)	n = 8; 5.6 (5.2, 6.3)	0.076
PROMIS (↑)	32.6 ± 8.5	n = 8; 23.8 ± 5.6	**0.015**
PC-PTSD-5 (↓)	1.0 (0.0, 2.0)	n = 9; 1.0 (0.0, 1.0)	0.827
EQ5D (↓)	7.0 (5.0, 8.5)	n = 9; 12.0 (10.0, 13.0)	**0.014**
CLCIC (↓)	5.0 (1.5, 8.0)	n = 9; 6.0 (4.0, 8.0)	0.610
USER-P (↑)	90.0 (75.8, 98.5)	n = 8; 61.6 (52.5, 78.3)	**0.017**
HADS Depression (↓)	2.0 (1.0, 3.5)	n = 8; 7.0 (5.0, 8.2)	0.080
HADS Anxiety (↓)	3.0 (2.0, 8.5)	n = 7; 1.0 (0.5, 4.5)	0.166
**CT Abnormalities**			
Ground-glass opacity/Consolidations	12/15 (80.0%)	8/11 (72.7%)	1.000
Bronchiectasis	5/15 (33.3%)	7/11 (63.6%)	0.233
Subpleural reticulation	4/15 (26.7%)	3/11 (27.3%)	1.000
Lymphadenopathy	3/15 (20.0%)	1/11 (9.1%)	0.614
Airtrapping	2/15 (13.3%)	1/11 (9.1%)	1.000
**Acute Phase Severity**			
Mild	0/15 (0.0%)	0/11 (0.0%)	1.000
Moderate	4/15 (26.7%)	2/11 (18.2%)	
Severe	11/15 (73.3%)	9/11 (81.8%)	
**Acute Phase Complications**			
Hospital duration (Days)	n = 14; 21.5 (10.8, 33.0)	53.0 (21.0, 59.5)	0.100
ICU admission	15/15 (100.0%)	11/11 (100.0%)	1.000
Pulmonary embolism	1/15 (6.7%)	5/11 (45.5%)	0.054
Thrombosis	3/15 (20.0%)	6/10 (60.0%)	0.087
Antibiotic use	10/15 (66.7%)	8/11 (72.7%)	1.000
**Dominant SARS-CoV-2 Virus Type**			
Alpha	4/15 (26.7%)	6/11 (54.5%)	0.399
Delta	9/15 (60.0%)	4/11 (36.4%)	
Omicron	2/15 (13.3%)	1/11 (9.1%)	

**Arrows indicate if a higher or lower score is more desirable for the patient.*

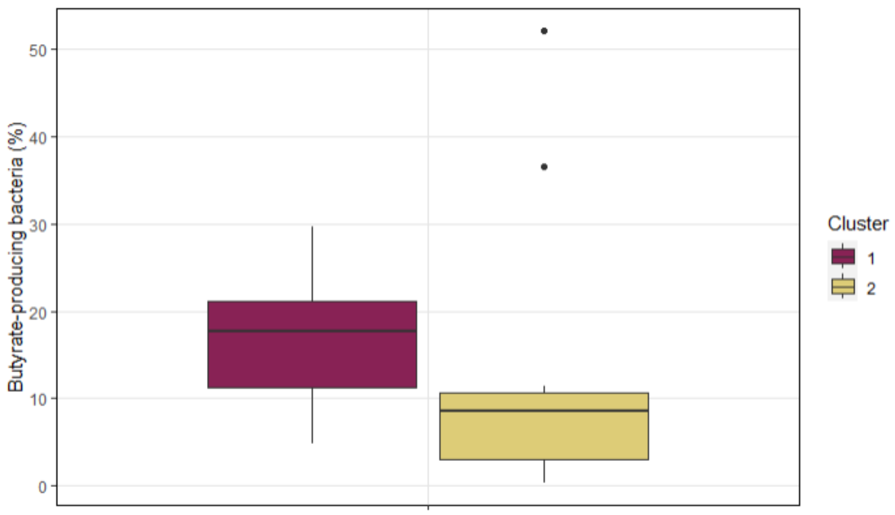

Figure S3 butyrate producers ICU admission*: Comparison of the proportion of butyrate producing bacteria between the clusters. Only patients admitted to the ICU are present in this figure.*
